# Virulence and Antimicrobial Resistance Profiling of *Salmonella* Serovars Recovered from Retail Poultry Offal in KwaZulu-Natal Province, South Africa

**DOI:** 10.3390/pathogens12050641

**Published:** 2023-04-25

**Authors:** Lindokuhle Ndlovu, Patrick Butaye, Tsolanku. S. Maliehe, Kudakwashe Magwedere, Bongi B. Mankonkwana, Albertus K. Basson, Siyanda. S. Ngema, Evelyn Madoroba

**Affiliations:** 1 Department of Biochemistry and Microbiology, University of Zululand, KwaDlangezwa 3886, South Africa; 201814448@stu.unizulu.ac.za (L.N.); 201732875@stu.unizulu.ac.za (B.B.M.); bassona@unizulu.ac.za (A.K.B.); 201100298@stu.unizulu.ac.za (S.S.N.); 2 Department of Pathobiology, Pharmacology and Zoological Medicine, Faculty of Veterinary Medicine, Salisburylaan 133, 9820 Merelbeke, Belgium; patrick.butaye@ugent.be; 3 Department of Water and Sanitation, University of Limpopo, Polokwane 0727, South Africa; tsolanku.maliehe@ul.ac.za; 4 Directorate of Veterinary Public Health, Department of Agriculture, Land Reform and Rural Development, Pretoria 0001, South Africa; kudakwashem@dalrrd.gov.za

**Keywords:** *Salmonella* serovars, virulence genes, antimicrobial resistance, poultry offal

## Abstract

As poultry organ meat is widely consumed, especially in low- and middle-income countries, there is reason to investigate it as a source of *Salmonella* infections in humans. Consequently, the aim of this study was to determine the prevalence, serotypes, virulence factors and antimicrobial resistance of *Salmonella* isolated from chicken offal from retail outlets in KwaZulu-Natal, South Africa. Samples (*n* = 446) were cultured for the detection of *Salmonella* using ISO 6579-1:2017. Presumptive *Salmonella* were confirmed using matrix-assisted laser desorption ionisation time-of-flight mass spectrometry. *Salmonella* isolates were serotyped using the Kauffmann–White–Le Minor scheme and antimicrobial susceptibility was determined by the Kirby–Bauer disk diffusion technique. A conventional PCR was used for the detection of *Salmonella invA*, *agfA*, *lpfA* and *sivH* virulence genes. Of the 446 offal samples, 13 tested positive for *Salmonella* (2.91%; CI = 1.6–5). The serovars included *S.* Enteritidis (*n* = 3/13), *S*. Mbandaka (*n* = 1/13), *S.* Infantis (*n* = 3/13), *S*. Heidelberg (*n* = 5/13) and *S*. Typhimurium (*n* = 1/13). Antimicrobial resistance against amoxicillin, kanamycin, chloramphenicol and oxytetracycline was found only in *S*. Typhimurium and *S*. Mbandaka. All 13 *Salmonella* isolates harboured *invA*, *agfA*, *lpfA* and *sivH* virulence genes. The results show low *Salmonella* prevalence from chicken offal. However, most serovars are known zoonotic pathogens, and multi-drug resistance was observed in some isolates. Consequently, chicken offal products need to be treated with caution to avoid zoonotic *Salmonella* infections.

## 1. Introduction

In South Africa, chicken production is the fastest growing animal protein industry and accounted for approximately 39.9% of the animal product gross value and 16.6% of the total agricultural gross value in 2021 [1]. Chicken meat is relatively cheap, which makes it affordable in many less resourced communities [2]. However, poultry meat may be contaminated along the value chain by foodborne pathogens such as non-typhoidal *Salmonella* (NTS), resulting in salmonellosis in consumers. There are an estimated 1.35 million non-typhoidal *Salmonella* infections in the United States each year [3]. As NTS infections are self-limiting in most cases, invasive infections are less frequent and are estimated at 26,500 hospitalizations and 420 fatalities on an annual basis [3]. Invasive infections are mainly seen in persons with underlying diseases such as malaria, sickle cell anaemia and HIV as well as malnourishment [4,5].

While contaminated food is the major route of transmission for NTS, the bacteria may be transmitted via products contaminated with faecal material by direct contact with animals that themselves are contaminated with faecal material and, on rare occasion, from person-to-person [6]. Animal source products are mainly involved, and about 20% of the cases are associated with poultry products [7]. The cross-contamination of foods is also an important factor increasing the risk of infections [5]. In South Africa, food laws such as the Meat Safety Act (Act No. 40 of 2000) and the Food Stuffs, Cosmetics and Disinfectants Act (Act No. 54 of 1972) guide the hygiene and quality assurance of food of animal origin. Despite these acts, the transmission of *Salmonella* continues to be a threat because the contamination of food is a complicated process [8].

Antimicrobial resistance (AMR) is when a micro-organism acquires the ability to resist the effect of antimicrobials. Resistance is selected when using antimicrobials; hence, abandoning the excessive use of antibiotics for growth promotion and preventive use can partly mitigate the challenge [9]. The mechanism of AMR is mediated by mutations or by the acquisition of specific resistance genes, and the latter are potentially transferable via the conjugation of mobile genetic elements, transduction or transformation [10]. While the Medicines and Related Substances Control Act (Act 101) prohibits access to antimicrobials without prescription in South Africa, the Farm Feeds, Agriculture Remedies, Fertilizers and Stock Remedies Act (Act No, 36 of 1947) provides for the over-the-counter purchase of antimicrobials by farmers for animal treatment [11].

Most studies on *Salmonella* in poultry product categories are on poultry carcasses, while only some have been on offal [12,13,14]. Furthermore, these products have a risk of being contaminated with faecal material, containing NTS. Gizzards are, moreover, part of the gastrointestinal tract and may be more prone to be contaminated via regurgitation or the seeping in of the water from the defeathering bath as well as the ingestion of feed contaminated with faecal material. Antimicrobial resistance is important to investigate for eventual therapies in humans, and the detection of virulence genes confirms the pathogenic potential. Therefore, this study aimed to determine the prevalence, serotypes, antimicrobial resistance and certain characteristics of *Salmonella* in chicken offal from some retail outlets located in the province of KwaZulu-Natal, South Africa.

## 2. Materials and Methods

### 2.1. Ethical Clearance

This study was approved by the University of Zululand’s Research Ethics Committee (UZREC) with ethics approval reference UZREC 171110-030 Dept 2022/11.

### 2.2. Study Area

**Figure 1:**
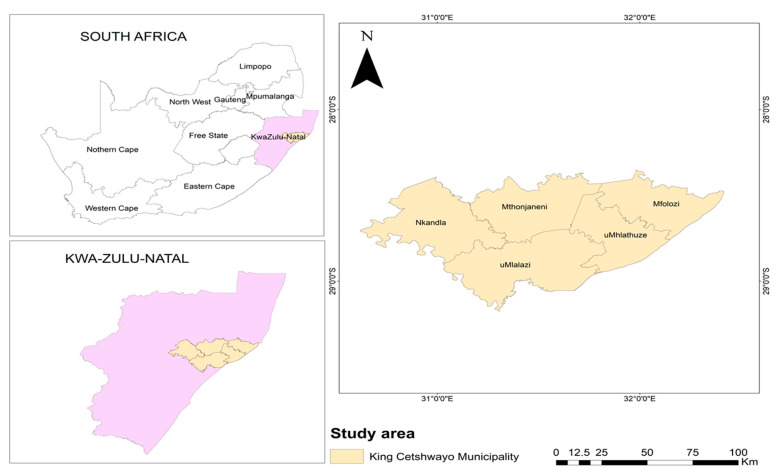
Map of King Cetshwayo District in KZN, South Africa. Source: Municipal Directory of South Africa [16].

Offal samples (*n* = 446) were purchased from 10 retail outlets in King Cetshwayo District in KZN, South Africa (Figure 1). King Cetshwayo district covers an area of 8214 km^2^ in the north coast region of KZN [15].

### 2.3. Study Design

The sample collection was based on the convenience and proximity of retail outlets in King Cetshwayo District. The sample size was determined using an equation for cross-sectional studies [17]:

$$n=\frac{Z{\left(1-\frac{\propto }{2}\right)}^{2}p\left(1-p\right)}{d^{2}}$$

where

*n* = sample size;

*Z* ${\left(1-\frac{\propto }{2}\right)}^{2}$ = the standard normal variate (at 5% type 1 error, yielding 1.96);

*p* = the expected proportion in the population based on previous studies;

*d* = the absolute error.

There are few studies on *Salmonella* in poultry offal that are published, and the differences in prevalence are high [12,13,14,18]; therefore, the estimated prevalence of 50% was used in this study. Using this estimated prevalence, the minimum sample size of 384 was calculated. In this study, 446 samples were used.

### 2.4. Sample Collection

Sampling was conducted from February 2022 to October 2022. The samples included raw chicken gizzards (*n* = 284), chicken hearts (*n* = 90) and chicken livers (*n* = 72). The samples were taken aseptically and packed individually in sterile polyethylene bags and transported on ice to the University of Zululand, Kwa-Dlangezwa campus. We sampled 10 retail outlets, which were at least 25 km from each other. In that area, there are also diverse poultry farms. As such, we assume that there are few to no samples originating from the same farm.

### 2.5. Microbiological Analysis

#### 2.5.1. Reference Strains for Quality Control

*Salmonella* Typhimurium ATCC 14028 was used as positive control and *Escherichia coli* ATCC 25922 was used as negative control in the isolation protocols. Field strains harbouring the *invA*, *agfA*, *lpfA* and *sivH* virulence factors from a previous study were included as control strains [19].

#### 2.5.2. Isolation and Identification of *Salmonella*

Isolation of *Salmonella* was conducted using International Organization for Standardization (ISO) 6579-1:2017. For pre-enrichment, 25 g of offal sample was homogenized in 225 mL of buffered peptone water (Oxoid Ltd., Basingstoke, UK), followed by incubation at 35 ± 2 °C for 18 ± 2 h. After incubation, the selective enrichment involved inoculation of 1 mL and 0.1 mL of pre-enriched suspensions into 10 mL of Mueller Kauffmann tetrathionate novobiocin (MKTTn) broth (Oxoid Ltd., Basingstoke, UK) and Rappaport-Vassiliadis Soya (RVS) broth (Merck, Darmstadt, Germany), respectively. Inoculated MKTTn and RVS broths were incubated for 24 ± 2 h at 35 ± 2 °C and 41.5 ± 2 °C, respectively. Each MKTTn and RVS broth suspension was streaked onto Xylose Lysine Deoxycholate (XLD) agar (Oxoid Ltd., Basingstoke, UK) and Bismuth Sulphite (BS) agar (Condalab laboratories, Madrid, Spain), followed by incubation at 35 ± 2 °C for 24 ± 2 h. Presumptive *Salmonella* colonies on XLD appeared to be pink with black centre. On BS, presumptive *Salmonella* colonies appeared as brown, grey or black with metallic sheen. Three presumptive *Salmonella* per sample were preserved in nutrient broth (Oxoid Ltd., Basingstoke, UK) supplemented with glycerol (30% final concentration) until required for further analysis. The preserved isolates were stored at −20 ± 2 °C. After storage, presumptive *Salmonella* were grown and purified again on nutrient agar plates (Oxoid Ltd., Basingstoke, UK). Presumptive *Salmonella* were identified using MALDI-TOF MS technology [20,21,22]. One isolate per sample was used for further downstream analysis.

#### 2.5.3. *Salmonella* Serotyping

The *Salmonella* strains were serotyped according to Kauffman–White–Le Minor classification scheme [23] using slide agglutination [24].

#### 2.5.4. Antimicrobial Susceptibility Testing

Antimicrobial susceptibility was determined using Kirby–Bauer disk diffusion method and interpretation was performed according to Clinical and Laboratory Standards Institute (CLSI) guidelines [25]. The *Salmonella* bacterial suspensions were prepared in physiological saline and the turbidity of each suspension was adjusted to an equivalent of 0.5 McFarland standard, which was verified via measurement of optical density (0.08–0.13) at 625 nm using a spectrophotometer (Merck Spectroquant Pharo, Darmstadt, Germany). Sterile cotton swabs were dipped into tubes containing the *Salmonella* suspensions and excess fluid was removed. The swabs were then inoculated onto the surface of Mueller Hinton agar (Thermofisher, Waltham, MA, USA) to ensure even distribution and confluent growth. The plates were left to dry for approximately 5 min at room temperature prior to insertion of antimicrobial disks on the inoculated agar. The following antibiotics were tested: ampicillin (10 µg), ciprofloxacin (5 µg), cefoxitin (30 µg), kanamycin (30 µg), oxytetracycline (30 µg), chloramphenicol (30 µg) and cefotaxime (30 µg) (Mast Laboratories, Bootle, UK). The selection criteria was based on the presence of breakpoints in the CLSI (2021) and/or purported use of the antimicrobial during poultry production. The inoculated plates were incubated at 35 °C ± 2 °C for 16 to 18 h. The inhibition zone diameters were measured using a ruler and results were interpreted according to Clinical Laboratory Standards (2021) as sensitive (S), intermediate (I) or resistant (R). *Escherichia coli* ATCC 25922 and *Salmonella* Typhimurium ATCC 14028 were included as quality control strains.

### 2.6. Detection of Salmonella Virulence Genes Using Polymerase Chain Reaction (PCR)

Genomic DNA was extracted from the *Salmonella* cultures using the Quick-DNA™ Fungal/Bacterial Miniprep Kit (Zymo Research, CA, USA, Catalogue No. D6005) according to the manufacturer’s instructions.

PCR amplification was performed to determine the presence of the virulence factor genes, including *invA*, *agfA*, *lpfA* and *sivH*, as described by Siddiky et al. [26], with slight modifications, and *Salmonella* strains from a previous study that tested positive for these genes were used as controls. The primers used for PCR amplification and the amplicon sizes are shown in Table 1. Each of the 20 µL PCR reactions consisted of NEB OneTaq 2X MasterMix with standard buffer (Inqaba Biotech, Pretoria, South Africa); genomic DNA (10–30 ng/μL; 1 µL); forward primer (10 μM; 1 µL); reverse primer (10 μM; 1 µL); and nuclease free water to make up the final volume of 20 µL. PCR thermocycling conditions were as follows: denaturation at 94 °C for 5 min, followed by 35 cycles of denaturation at 94 °C for 30 s, 50 °C for 30 s and 68 °C for 1 min. Final extension of PCR amplicons was carried out at 68 °C for 10 min. Agarose gel electrophoresis was conducted using 1.5% agarose gels with ethidium bromide and run at 3 Volts/cm for 1 h as previously described [19]. Estimation of the PCR amplicon sizes was performed using a 100 bp DNA ladder (Inqaba Biotech, Pretoria, South Africa). Gels were viewed under ultraviolet light, and documentation was performed using a gel documentation system (E-Box; Vilber, Eberhardzell, Germany).

### 2.7. Statistical Analysis

Confidence intervals (CI) for the population proportions were calculated with a lower and higher bound using exact binomial distribution in Excel [27]. Chi square test was used to calculate differences between offal types.

## 3. Results

### 3.1. Prevalence of Salmonella from Chicken Offal

Table 2 shows the summary of the prevalence of *Salmonella* in the different chicken offal samples. Out of the 446 chicken offal samples, 13 samples tested positive for *Salmonella* species. No significant difference was found between the offal types.

### 3.2. Salmonella Serotypes

We found three *S.* Enteritidis strains, five *S*. Heidelberg, three *S*. Infantis, one *S*. Mbandaka and one *S*. Typhimurium in the offal. All serotypes detected were found in the gizzard samples, while the sole positive heart sample was contaminated with *S*. Enteritidis.

### 3.3. Antimicrobial Resistance of Salmonella

Out of the 13 strains, only 1 *S*. Typhimurium and 1 *S*. Mbandaka showed resistance (Table 3). They had the same resistance profile as against amoxicillin kanamycin, chloramphenicol and oxytetracycline.

### 3.4. PCR for Detection of Virulence Genes

All tested genes were present in the 13 *Salmonella* isolates from this study.

## 4. Discussion

A relatively low *Salmonella* prevalence (2.9%) was observed in this study, and this prevalence is nearly the same as that determined in a former national *Salmonella* surveillance of poultry meat and meat products from different establishments in the nine provinces of South Africa [28]. This indicates that offal products are similarly contaminated compared to other poultry meat cuts. This is somehow logical as the cross-contamination of meat and offal occur similarly during successive slaughtering steps.

Gizzards constituted the most positive samples; however, they were not significantly different from the other samples. A larger sample size of each sample is needed to eventually show if there are significant differences. However, it is not surprising to observe higher contamination in gizzards than in other offal (except intestines) as the gizzard is more likely to be exposed to *Salmonella* from the food and bedding. In addition, because it is part of the gastrointestinal system, it may be more prone to colonization with *Salmonella* or the seeping in of water from the defeathering bath. A recent study on ready-to-eat chicken gizzards in Nigeria showed a much higher *Salmonella* prevalence of 8.6% [29]; however, it is challenging to compare the study from Nigeria with findings from this study as the additional treatment or food safety hurdles might reduce or increase the *Salmonella* prevalence depending on the hygiene applied.

Two of the serotypes found in this study, namely, *S*. Enteritidis and *S*. Typhimurium, are the serotypes that have been most often reported in salmonellosis in humans worldwide [30]. Outbreaks in humans due to *S*. Heidelberg, *S*. Mbandaka and *S*. Infantis have also been associated with poultry products [3,31,32], though to a lesser extent. The *Salmonella* serovars in chicken offal meat highlight the potential risks of poultry offal to consumers. Therefore, the monitoring and surveillance of *Salmonella* should be implemented, and food hygiene awareness should be given to the consumers of these products to prevent, reduce and eliminate NTS infections.

All but two isolates were susceptible to the antimicrobial agents tested. The *S*. Typhimurium and the *S*. Mbandaka isolates were resistant to four antimicrobial agents of four different classes and are to be regarded as multi-drug resistant (amoxicillin, kanamycin, chloramphenicol and oxytetracycline) [33]. It should be noted, however, that antimicrobial resistance is serotype dependent. For example, *S.* Enteritidis is, in general, less resistant than many other serotypes found in poultry and on poultry products [34]. The presence of foods with multi-drug resistant strains of *Salmonella* is a threat to people consuming these food products; however, based on our results, the threat of these products in South Africa is not that large. This could be due to the self-limiting nature of NTS infection in general circumstances.

Four genes were chosen for assessing the virulence of the strains. The *invA* gene is associated with epithelial cell invasion and the recognition of the host [8,19,35,36]. The *agfA* gene encodes fimbriae, which is important for attachment during pathogenesis, the enhanced survival of the *Salmonella* strains and improved eukaryotic cell invasion [37]. It is also involved in adhesion to diverse surfaces and in biofilm formation, which makes it a potential challenge in food production and handling establishments [38,39]. The *lpfA* gene forms part of an operon that encodes the long polar fimbriae and is associated with bacterial adhesion [36,38], which is important for the production of biofilms [40]. The *sivH* gene encodes the outer membrane protein, and its presence is linked to intestinal colonization [36]. The isolated strains are, thus, well equipped to cause disease.

A limitation of this study is that, despite the unofficial knowledge that chicken offal is consumed by different people in South Africa, we could not find official statistics of the consumption patterns by different groups. This information could guide strategies for food safety measures in addition to the currently existing information about previous findings in other poultry meat cuts. It is, therefore, paramount to undertake studies on the consumption patterns of chicken offal in South Africa and on consumer access to information about the safety of these products.

## 5. Conclusions

In this study, we found a low prevalence of *Salmonella* in chicken gizzard, heart and liver at retail level, which is consistent with the findings on other poultry meat samples and confirms findings from the literature. This indicates that these products should be handled using strict hygiene measures in the same way as chicken meat to avoid *Salmonella* infections in humans. Studies are needed to determine which offal products might be more contaminated as we isolated, albeit non-significantly, more strains from gizzard samples. This is important for the implementation of targeted control strategies during offal handling. The *Salmonella* strains were, in general, susceptible to tested antimicrobials; however, multi-resistant *Salmonella* strains were also found, which is a threat to people consuming poultry offal. The presence of all four tested virulence factors in all *Salmonella* strains suggests that the strains were well equipped to cause disease in humans and cause hygienic challenges along the food chain.

## Figures and Tables

**Table 1:** Primers used for PCR amplification and the amplicon sizes.

Target Gene	Primer Sequence (5′–3′)	Amplicon Size (bp)	Reference
*InvA*	F-GTGAAATTATCGCCACGTTCGGGCAA R-TCATCGCACCGTCAAAGGAACC	284	[26]
*AgfA*	F-TCCACAATGGGGCGGCGGCG R-CCTGACGCACCATTACGCTG	350	[26]
*LpfA*	F-CTTTCGCTGCTGAATCTGGT R-CAGTGTTAACAGAAACCAGT	250	[26]
*SivH*	F-GTATGCGAACAAGCGTAACAC R-CAGAATGCGAATCCTTCGCAC	763	[26]

**Table 2:** Summary of the prevalence of *Salmonella* in chicken offal from this study.

Offal Type	Number of Samples	Positive Samples	Prevalence (%)	CI
Gizzards	284	12	4.23	2.2–7
Hearts	90	1	1.1	0.03–6
Livers	72	0	0	0–5
Total	446	13	2.91	1.6–5

**Table 3:** Summary of antimicrobial susceptibility testing.

Sample ID	AP	K	CTX	FOX	CIP	C	OT
G180	S	S	S	S	S	S	S
169	S	S	S	S	S	S	S
H207	S	S	S	S	S	S	S
G398	R	R	S	S	S	R	R
G362	S	S	S	S	S	S	S
G444	S	S	S	S	S	S	S
G345	S	S	S	S	S	S	S
G343	S	S	S	S	S	S	S
G399	S	S	S	S	S	S	S
G352	S	S	S	S	S	S	S
G357	S	S	S	S	S	S	S
G307	R	R	S	S	S	R	R
127	S	S	S	S	S	S	S

S—sensitive; R—resistant; AP—Ampicillin; CIP—Ciprofloxacin; FOX—Cefoxitin; K—Kanamycin; OT–Oxytetracycline; C—Chloramphenicol; CTX—Cefotaxime.

## Data Availability

The datasets used during the current study are available from the corresponding author on reasonable request.
